# A Pilot Study of Circulating miRNAs as Potential Biomarkers of Early Stage Breast Cancer

**DOI:** 10.1371/journal.pone.0013735

**Published:** 2010-10-29

**Authors:** Hua Zhao, Jie Shen, Leonard Medico, Dan Wang, Christine B. Ambrosone, Song Liu

**Affiliations:** 1 Department of Cancer Prevention and Controls, Roswell Park Cancer Institute, Buffalo, New York, United States of America; 2 Department of Biostatistics, Roswell Park Cancer Institute, Buffalo, New York, United States of America; Baylor College of Medicine, United States of America

## Abstract

**Background:**

To date, there are no highly sensitive and specific minimally invasive biomarkers for detection of breast cancer at an early stage. The occurrence of circulating microRNAs (miRNAs) in blood components (including serum and plasma) has been repeatedly observed in cancer patients as well as healthy controls. Because of the significance of miRNA in carcinogenesis, circulating miRNAs in blood may be unique biomarkers for early and minimally invasive diagnosis of human cancers. The objective of this pilot study was to discover a panel of circulating miRNAs as potential novel breast cancer biomarkers.

**Methodology/Principal Findings:**

Using microarray-based expression profiling followed by Real-Time quantitative Polymerase Cycle Reaction (RT-qPCR) validation, we compared the levels of circulating miRNAs in plasma samples from 20 women with early stage breast cancer (10 Caucasian American (CA) and 10 African American (AA)) and 20 matched healthy controls (10 CAs and 10 AAs). Using the significance level of *p*<0.05 constrained by at least two-fold expression change as selection criteria, we found that 31 miRNAs were differentially expressed in CA study subjects (17 up and 14 down) and 18 miRNAs were differentially expressed in AA study subjects (9 up and 9 down). Interestingly, only 2 differentially expressed miRNAs overlapped between CA and AA study subjects. Using receiver operational curve (ROC) analysis, we show that not only up-regulated but also down-regulated miRNAs can discriminate patients with breast cancer from healthy controls with reasonable sensitivity and specificity. To further explore the potential roles of these circulating miRNAs in breast carcinogenesis, we applied pathway-based bioinformatics exploratory analysis and predicted a number of significantly enriched pathways which are predicted to be regulated by these circulating miRNAs, most of which are involved in critical cell functions, cancer development and progression.

**Conclusions:**

Our observations from this pilot study suggest that the altered levels of circulating miRNAs might have great potential to serve as novel, noninvasive biomarkers for early detection of breast cancer.

## Introduction

Discovery of sensitive and specific minimally invasive biomarkers that can be exploited to detect early neoplastic changes, thus facilitating the detection of breast cancer at an early stage, is one of the most important challenges in the management of breast cancer. The ideal biomarker should be easily accessible such that it can be sampled relatively noninvasively, sensitive enough to detect early presence of tumors in almost all patients, and absent or minimally present in healthy, tumor free individuals. Unfortunately, none of the existing diagnostic tools and biomarkers for breast cancer meets the above criteria [1]–[3]. For example, mammography, currently the gold standard diagnostic tool, needs to use ionizing radiation and has a false positive rate of 8% to 10% [1]. A number of circulating tumor markers, such as carcinoembryonic antigen and carbohydrate antigen 15–3, have shown promise in the management of breast cancer, but the sensitivity of these markers is low, and so they are not useful for the early detection of breast cancer [4]–[6]. Clearly, the development of minimally or noninvasive, highly sensitive and specific breast cancer biomarkers which can complement and improve on current strategies for breast cancer detection is urgently needed.

In the last decades, the relationship between microRNA (miRNA) and human cancer has been extensively investigated. Research has shown that miRNAs are deregulated in a widespread manner in almost every type of human cancer, and the signature of miRNA expression in tumors can be potentially used as biomarkers for tumor characterization and cancer prognosis [7]–[19]. Circulating RNAs have been identified in the serum/plasma of cancer patients for more than a decade. Recently, several studies have reported the occurrence of circulating miRNAs in serum and plasma samples from both cancer patients and healthy controls [20]–[28]. For example, in colorectal cancer, Huang *et al* [27] found plasma miRNAs were highly sensitive for detecting colorectal cancer and advanced adenomas, and that *miR-29a* and *miR-92a* were associated with advanced neoplasia. To further explore the origins of these circulating miRNAs, they compared the levels of *miR-29a* and *miR-92a* expression in plasma samples harvested from pre-operative and post-operative bloods and found a significant reduction in both miRNAs compared to the pre-operative samples of the same patients. In breast cancer, Heneghan *et al* [26] surveyed a panel of 7 candidate miRNAs in whole blood RNAs from 148 breast cancer patients and 44 age-matched and disease free controls. They found the expression of *miR-195* was significantly elevated in breast cancer patients. Additionally, they observed a significant reduction in *miR-195* in post-operative whole blood compared to the pre-operative samples of the same patients.

Using microarray-based expression profiling, the goal of this pilot study was to identify a panel of circulating miRNAs which are differentially expressed in plasma samples from breast cancer patients and matched healthy controls, and to examine if there were differences in circulating miRNA expression between Caucasians Americans (CAs) and African Americans (AAs). We also aimed to apply bioinformatics tools to explore the potential biological function of identified candidate miRNAs.

## Materials and Methods

### Study population

The study has been approved by Institutional Research Board (IRB) of Roswell Park Cancer Institute. Anonymized biospecimens and questionnaire data used in this study were made available through the Roswell Park Cancer Institute's (RPCI) Data Bank and BioRepository (DBBR) [29]. Patients are enrolled through site-specific clinics prior to surgery and/or chemotherapy, and controls are individuals who are free from cancer and are visitors or family members of patients. Relationships between patients and controls are carefully annotated, so that we avoid overmatching patients to their own family or friends. Written consent is obtained from every individual before he/she enrolls in the DBBR. The consent will allow DBBR to provide anonymized biospecimens and questionnaire data for research (such as this study) without further consent. Patients and controls are consented to provide a non-fasting blood sample and to complete a questionnaire. Blood samples are drawn in phlebotomy and transferred to the DBBR laboratory. Following DBBR standard operating procedure (SOP), samples are processed and blood components stored within one hour of collection to minimize degradation. Ten milliliters of whole blood was obtained from each study subject. Plasma was extracted by centrifuging whole blood at 3,000 rpm for 10 minutes at room temperature. All extracted plasma samples are stored in phased liquid nitrogen. To minimize the effect of freeze-thaw on circulating miRNAs, we only used plasma samples which had not been previously thawed. In this study, a total of 20 women with breast cancer and 20 cancer-free women were included in the microarray profiling analysis. Same AA study participants (10 AA cases and 10 AA controls) were included in the RT-qPCR validation analysis. The CA study participants (15 CA cases and 15 CA controls) included in RT-qPCR validation analysis were also obtained from DBBR, but they were different from the ones used in microRNA profiling.

### RNA isolation

Total RNA, including miRNA from plasma, was isolated using the miRNeasy kit (Qiagen) with minor modifications. In brief, 700 µl of QIAzol reagent was added to 200 µl of plasma sample. The sample was mixed in a tube, followed by the addition of 3 µl of miSPIKE, spiked-in miRNA, at a concentration of 0.1 µM (IDT) and 140 µl of chloroform. After mixing vigorously for 15 s, the sample was then centrifuged at 12,000 g for 15 minutes. The upper aqueous phase was carefully transferred to a new collection tube, and 1.5 volume of ethanol was added. The sample was then applied directly to a silica membrane-containing column and the RNA was bound and cleaned using buffers provided by the manufacturer to remove impurities. The immobilized RNA was then collected from the membrane with a low salt elution buffer. The quality and quantity of the RNA was evaluated by 260/280 ratio using NanoDrop spectrophotometry (NanoDrop ND-1000 Technologies Inc.) and Agilent 2100 Bioanalyzer (Agilent Technologies). The efficiency of small RNA isolation was monitored by the amount of spiked-in miRNA recovered by using PCR with sequence specific primers (IDT).

### MicroRNA Quantification of miRNA Expression

Two hundred ng of total RNA from each sample were labeled and hybridized on Human v2 MicroRNA Expression BeadChips (Cat. no. MI-102–1024; Illumina), according to the manufacturers recommendations (Illumina MicroRNA Expression Profiling Assay Guide). The raw intensity of Illumina Plasma V2 MicroRNA expression array was scanned and extracted using BeadScan, with the data corrected by background subtraction in GenomeStudio module. The lumi package in the R-based Bioconductor Package was used to normalize the log2 transformed intensity data with the Quantile normalization algorithm. The expression profiles have been deposited in NCBI's Gene Expression Omnibus (GEO) with accession number GSE22981.

### microRNA MicroArray Data Analysis

All data analysis was performed under R programming environment (www.r-project.org). *Differential Expression Testing* For the 40 subjects, we compared cases and controls stratified by race/ethnicity. We used the Limma program in the R-based Bioconductor package to calculate the level of differential expression for each comparison. Briefly, for each comparison, a linear model was fit to the data (with cell means corresponding to the different conditions and a random effect for array) [30]. For each comparison, we obtained the list of differentially expressed microRNA constrained by P-value less than 0.05 and then checked for candidates with at least two-fold expression change. *Clustering* Following single miRNA-based significance testing, we used the expression value of miRNAs (*P value* <0.05 and at least two-fold expression) to cluster the patients for each comparison. Our purpose was to check whether the identified miRNAs for each comparison, as a whole, were able to serve as potential miRNA signature to classify patients into their corresponding case/control status. Hierarchical clustering based on the average linkage of Pearson Correlation was employed [31]. The expression value of the same miRNA lists was also used as the input for Principal Component Analysis (PCA). Briefly, the 1st principal component (i.e., the direction along which the miRNAs show largest variation) and the 2nd principle component (i.e., the direction uncorrelated to the 1st component along which the miRNAs show the largest deviation) were shown to capture the clustering structures. The PCA implementation here is based on the single value decomposition (SVD) package. *ROC curve analysis* The expression profile of each identified miRNA was used as the input for Receive Characteristics Curve (ROC) analysis with the ROCR package [32]. ROC curve is displayed as the True Positive Rate (TPR) versus the False Positive Rate (FPR). The area under the ROC curve (AUC), a measure of discrimination accuracy, is reported. *Pathway Enrichment Analysis* Using TargetScan (http://www.targetscan.org), we obtained the list of genes predicted to be targeted by the miRNAs identified from each comparison. The predicted miRNA target genes were analyzed for enriched KEGG pathways by using the NCBI DAVID server (http://david.abcc.ncifcrf.gov) with default setting, in which the null hypothesis is that no difference exists between the number of genes falling into a given pathway in the target gene list and the genome as a whole [33].

### Real-time quantitative PCR analysis

The expression levels of miRNA were confirmed with a Taqman-based real-time quantitative PCR (RT-qPCR) using individual miRNA-specific primers and probes as described by the manufacturer (Applied Biosystems). The first-strand miRNA-cDNA PCR template was generated from 50 ng of total RNA according to the manufacturer's instructions. Approximately 2.5 ng of cDNA was then used in the PCR on a StepOnePlus Real-Time PCR System from Applied Biosystems. Triplicate samples, validated endogenous controls, and inter-assay controls were used throughout. The RT-qPCR results were analyzed by SDS 2.2.2. For the AA group, let-7d* and miR-425* were chosen for RT-qPCR validation (Table S2). For the CA group, *let-7c* and *miR-589* were chosen for RT-qPCR validation (Table S3, S4). So far, there is no reliable endogenous control microRNA in studying circulating microRNAs. We have surveyed the literature and found that expression of *miR-16* is relatively stable. Therefore, in this study, we chose *miR-16* as the endogenous control. RT-qPCR data were the normalized expression values in which the endogenous control *miR-16* was used as the reference gene. For each assay, the Ct (Cycle threshold) of miRNA of interest in the TaqMan qPCR assay was subtracted from the average *miR-16* Ct value to obtain a ΔCt value (*miR-16* - miRNA of interest). A higher delta Ct value indicates a higher expression level of the miRNA of interest.

## Results

### Demographic and clinical characterization of study population

Twenty breast cancer cases (10 CAs and 10 AAs) and 20 healthy controls (10 CAs and 10 AAs) were included in the analysis. The cases and controls were well-matched on age (mean age: 56 vs 57, P = 0.562). All cases had histologically confirmed early stage (I and II) invasive ductal carcinoma. Blood samples were drawn prior to surgery. The tumor size ranged between 0.2 to 2.5 cm. ER, PR and HER2/*neu* status data were available for 18 patients: 11 ER+, 7 ER−; 10 PR+, 8 PR−; and 6 HER2/*neu*+, 12 HER2/*neu−*. Four patients had triple negative breast cancer.

### A large number of miRNAs are detected in plasma

The expression-detection *P-*value of Illumina Expression BeadChips was used to quantify the number of miRNA detected in the plasma of participants, designed to test the null hypothesis that the expression intensity of a given miRNA is indistinguishable from the background intensity. As shown in *Table S1*, among a total of 1,145 miRNAs profiled by the chips, among CAs,there were 886, 518 and 266 miRNAs detected (with detection Pvalue<0.05) in the plasma of at least 1, 5, and 10 controls, respectively. The corresponding number for case subjects in CA group was 873, 503 and 278, respectively. Similar number of miRNAs was detected in the plasma of AA participants (*Table S1*). Overall, this demonstrates that plasma contains a large amount of detectable miRNAs which provide a valuable repertoire that can be used to discover circulating miRNA-based biomarkers for breast cancer early detection.

### Identification of differentially expressed miRNAs

**Figure 1:**
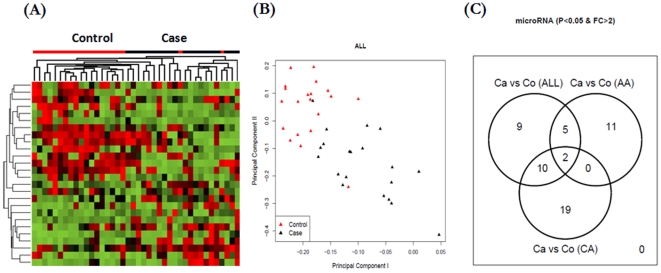
Characteristics of differentially expressed microRNAs (P<0.05 & FC >2) obtained from the case-versus-control comparison using all 40 participants. A–B) Hierarchical clustering and principal component clustering of differentially expressed microRNAs C) The overlap of differentially expressed microRNAs obtained from all 40 participants with those from AA group only (20 participants) and CA group only (20 participants), respectively.

At first, we made case-versus-control comparisons using all 40 participants (*i.e.*, 20 *vs.* 20). We identified 26 miRNAs with at least two-fold differential expression at the significance level of *p*<0.05 (Table 1), which reasonably separate the 20 cases from the 20 controls (Figure 1A–B). Compared with the differentially expressed miRNAs derived from case-versus-control comparisons stratified by race, 10 of these 26 miRNAs can be derived using CA group only, while 5 miRNAs can be derived using AA group only (Figure 1C). There are 11, 19 and 9 miRNAs unique to the comparison using AA group only, CA group only, and all 40 participants, respectively (Figure 1C). A total of 39 miRNAs are specific to one of the three groups, compared with the 15 miRNAs shared by two groups and 2 miRNAs shared by three groups, suggesting a potential racial difference in circulating miRNAs. Clearly, separate case-versus-control comparisons stratified by race are necessary.

**Table 1:** Differentially expressed microRNAs (P<0.05) with at least two-fold change obtained from case-versus-control comparisons in specimens of all 40 participants.

All participants (Case vs. Control)			
MicroRNA name	Log2 FC	P value	AUC
hsa-miR-595	2.395719	0.002393	0.75
hsa-miR-589	2.155282	0.006985	0.6
hsa-miR-504	1.915404	0.025783	0.68
hsa-miR-518b	1.600464	0.035285	0.67
hsa-miR-483-5p	1.385806	0.037231	0.56
hsa-miR-425*	1.197242	0.027131	0.68
hsa-miR-493	1.144993	0.032888	0.7
hsa-miR-187	1.144919	0.038317	0.62
hsa-miR-431*	1.107432	0.025801	0.62
hsa-miR-1231	1.028423	0.023928	0.68
solexa-9655-85	1.003729	0.026531	0.7
hsa-miR-668	−1.00174	0.038456	0.68
hsa-miR-377	−1.08111	0.048523	0.66
hsa-miR-410	−1.17687	0.039992	0.64
hsa-miR-922	−1.24073	0.029972	0.64
hsa-miR-155	−1.26546	0.014117	0.72
HS_169	−1.29076	0.023019	0.69
hsa-miR-340*	−1.50691	0.019858	0.66
HS_200	−1.53419	0.049367	0.7
hsa-miR-432	−1.60309	0.047606	0.65
hsa-miR-574-3p	−1.66389	0.037904	0.67
hsa-miR-148a	−1.68157	0.034794	0.66
hsa-miR-181a	−2.00397	0.004354	0.72
hsa-miR-1275	−2.00526	0.008116	0.72
hsa-miR-1304	−2.51079	0.002657	0.7
hsa-miR-151-5p	−2.81719	0.000542	0.76

In the case-versus-control comparison in CA group, we identified 31 miRNAs with at least two-fold differential expression at the significance level of *p*<0.05, with 17 miRNAs up-regulated in cases and 14 miRNAs down-regulated. Applying the same criteria to the AA group, we identified 18 differentially expressed miRNAs, with 9 miRNAs up-regulated in cases and 9 miRNAs down-regulated. The number of identified differentially expressed miRNAs (*p*<0.05) and the subgroup restricted by desired fold change for each comparison are summarized in Table 2. A detailed list of differentially expressed miRNAs with at least two-fold expression change is shown in Table 3.

**Table 2:** Summary of the number of differentially expressed miRNAs obtained from comparisons (case vs. control) in specimens from AA and CA participants, respectively.

	Case vs. Control		
	AA group	CA group	Overlap
Number of Patients	10 vs. 10	10 vs. 10	
Number of DEmRs (P<0.05)	23(10∧/13*)	36(20/16)	2
Number of DEmRs (P<0.05 & > = 1.5 fold change)	22(10/12)	34(18/16)	2
Number of DEmRs (P<0.05 & > = 2 fold change)	18(9/9)	31(17/14)	2

∧ : Up-regulated in the case vs control comparison.

*: Down-regulated in the case vs control comparison.

**Table 3:** Differentially expressed microRNAs (P<0.05) with at least two-fold change obtained from comparisons in specimens from AA and CA participants, respectively.

AA group (Case vs. Control)				CA group (Case vs. Control)			
MicroRNA Name	Log2 FC	P value	AUC	MicroRNA Name	Log2 FC	P value	AUC
HS_242	2.522	0.043	0.73	hsa-miR-504	3.0135	0.014	0.76
hsa-miR-425*	2.313	0.003	0.79	HS_217	2.9009	0.012	0.78
hsa-miR-483-5p	1.992	0.034	0.61	hsa-miR-589	2.7814	0.013	0.62
hsa-miR-485-3p	1.977	0.016	0.61	solexa-578-1915	2.7638	0.009	0.84
hsa-miR-431	1.784	0.041	0.76	hsa-miR-595	2.6937	0.014	0.77
HS_183.1	1.7	0.037	0.69	hsa-miR-608	2.6538	0.015	0.76
hsa-miR-493	1.481	0.050	0.7	hsa-miR-219-5p	2.1339	0.04	0.77
hsa-miR-558	1.426	0.043	0.73	hsa-miR-1	1.8684	0.035	0.68
hsa-miR-331-5p	1.156	0.032	0.8	hsa-miR-431*	1.8289	0.01	0.77
hsa-miR-409-5p	−1.007	0.012	0.64	hsa-miR-448	1.6971	0.025	0.6
hsa-miR-642	−1.184	0.033	0.7	solexa-826-1288	1.6054	0.015	0.73
hsa-miR-505	−1.572	0.017	0.58	hsa-miR-378	1.506	0.03	0.59
hsa-miR-377	−1.591	0.041	0.75	solexa-9655-85	1.4611	0.023	0.78
HS_257	−1.802	0.005	0.62	hsa-miR-551a	1.3358	0.041	0.74
hsa-miR-340*	−1.85	0.041	0.63	hsa-miR-302b*	1.2173	0.028	0.86
hsa-miR-181a	−2.054	0.034	0.69	hsa-miR-890	1.1176	0.024	0.78
hsa-miR-1304	−2.624	0.023	0.76	hsa-miR-548l	1.0465	0.022	0.83
hsa-let-7d*	−2.721	0.031	0.73	hsa-miR-873	−1.013	0.04	0.52
				hsa-miR-502-3p,hsa-miR-500*	−1.276	0.041	0.6
				hsa-miR-668	−1.405	0.04	0.77
				hsa-miR-610	−1.458	0.016	0.66
				hsa-let-7c	−1.568	0.015	0.84
				hsa-miR-155	−1.684	0.02	0.74
				hsa-miR-331-3p	−1.831	0.03	0.5
				hsa-miR-181a	−1.954	0.043	0.76
				hsa-miR-27b	−1.994	0.021	0.67
				hsa-miR-1304	−2.397	0.036	0.62
				hsa-miR-574-3p	−2.463	0.03	0.8
				hsa-miR-1275	−2.629	0.014	0.77
				hsa-miR-654-5p	−2.758	0.021	0.82
				hsa-miR-151-5p	−3.522	0.002	0.76

The common microRNAs are underlined.

### Characteristics of differentially expressed miRNAs

Remarkably, we found that there is little overlap between the differentially expressed miRNAs identified from the CA groups and those from the AA groups (Table 2–3). Only two of the 31 CA-derived miRNAs were also found in the 18 AA-derived miRNAs, namely miR-181a and miR-1304. These results, while preliminary, suggest that it might be necessary to develop race-specific circulating miRNA based biomarkers in breast cancer early detection.

**Figure 2:**
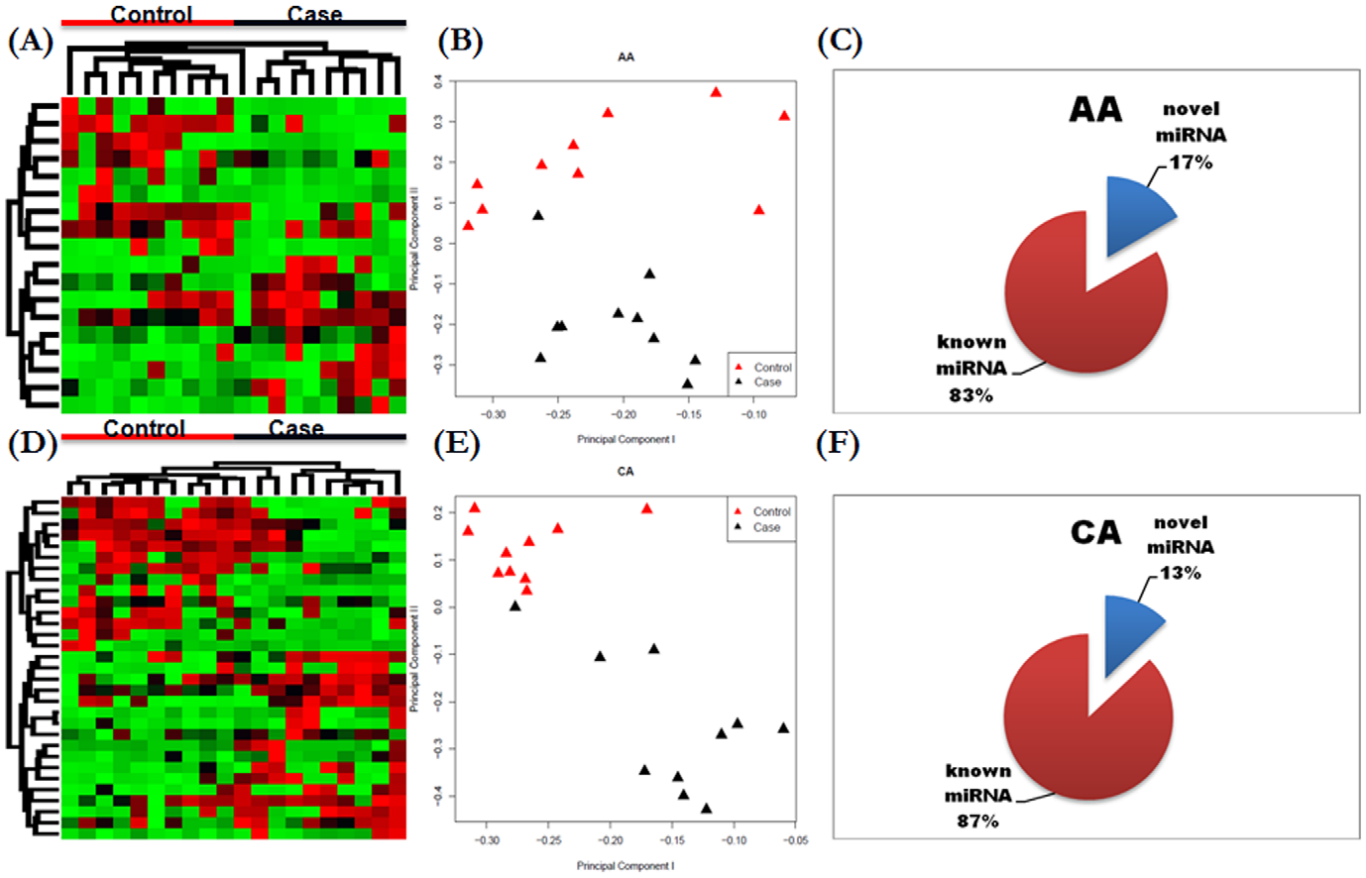
Characteristics of differentially expressed microRNAs (P<0.05 & FC >2) obtained from the case-versus-control comparison. A–B) Hierarchical clustering and principal component clustering of miRNAs in samples from AA participants. D–E) Hierarchical clustering and principal component clustering of miRNAs in samples from CA participants. C, F) The distribution of novel vs. known differentially expressed miRNAs obtained from AA group and CA group, separately.

The expression values of differentially expressed miRNAs identified from each comparison were used to cluster the participants into their corresponding case/control status. As shown in Figure 2A–B, the 18 differentially expressed miRNAs derived from the AA group reasonably separate the 10 cases from the 10 controls. Similarly, the 31 differentially expressed miRNAs derived from the CA group can reasonably separate the 10 cases from the 10 controls (Figure 2D–E). The clustering results demonstrate that the lists of differentially expressed miRNAs derived from case-versus-control study might collectively serve as potential circulating miRNA-based biomarkers in breast cancer early detection.

The Illumina Human v2 MicroRNA Expression BeadChips contains 1,145 miRNAs including 858 known miRNAs annotated by miRBase (http://www.mirbase.org/) and 287 novel miRNAs not found by miRBase (i.e., obtained from deep sequencing in human tissues http://www.illumina.com/). In both AA and CA groups, we found that novel miRNA constitute ∼15% of identified differentially expressed miRNAs (Figure 2C, 2F). For example, the serum level of *HS_242* and *HS_217* are the first and second most elevated in case *vs.* control in AA group and CA group, respectively. As the rapidly developingd next-generation sequencing technique provides unprecedented power to discover and characterize new miRNAs [34]–[35], it is expected that there will be more currently-unfound circulating miRNA whose biomarker potential remains to be explored.

### Exploratory *in silico* pathway analysis

**Figure 3:**
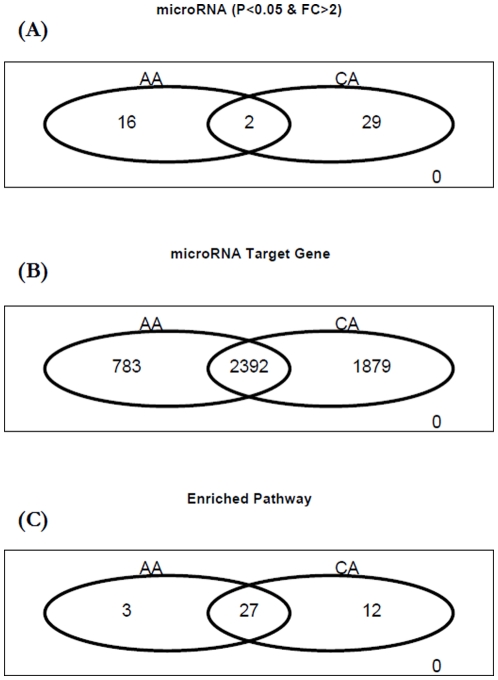
Venn diagrams showing the overlap at microRNA, target gene and pathway levels, respectively. **A**) The differentially expressed microRNAs (P<0.05 & FC >2). **B**) The target genes predicted to be regulated by differentially expressed microRNAs. **C**) The enriched pathways (P<0.01) in target genes predicted to be regulated by differentially expressed microRNAs.

As the number of experimentally validated miRNA targets is limited, we used the widely used TargetScan algorithm to obtain the list of Entrez genes predicted to be targeted by the miRNAs obtained from the AA study and CA study, respectively. We then use NCBI DAVID server to identify the significantly enriched canonical pathways (P<0.01) in these conserved targets. As shown in Figure 3, although there are few overlaps at the individual miRNA level, there is a much higher degree of convergence at the pathway level regulated by identified miRNAs. Specifically, 27 of 30 enriched pathways regulated by AA-derived miRNAs are also enriched in the predicted targets of CA-derived miRNAs.

As shown in Table 4, *pathways in cancer* (KEGG: hsa05200) is one of the most enriched pathways among the genes predicted to regulated by AA-derived (*P* = 3.78e^-09^) and CA-derived (*P* = 1.20^e-13^) miRNAs, respectively. This reassures the correctness of our approach and indicates the potentially important functional role of circulating miRNAs in tumor involvement. Pathways involved in various signal transduction and cell-cell interactions such as *ErbB signaling pathways*, *Focal adhesion*, and *Adherens junction* are also significantly enriched in both AA and CA case-control comparisons. Taken together, these exploratory analyses suggest that variation in the plasma level of key circulating miRNAs might affect critical pathways involved in breast cancer formation and progression, an important mechanism warranting follow-up research. As miRNA target prediction algorithm is known to contain both false positives and false negatives, and our *in silico* pathway enrichment analysis is based on mRNA genes predicted to be targeted by circulating miRNAs, a full understanding of the potential functional role of circulating miRNAs can only be established using functional experiments.

**Table 4:** The list of enriched pathways (P<0.01) in the genes predicted to be targeted by differentially expressed microRNAs (P<0.05) with at least two-fold change obtained from comparisons of case vs. control in AA and CA study subjects, respectively.

AA group (Case vs. Control)		CA group (Case vs. Control)	
Pathway Name	P value	Pathway Name	P value
hsa04010:MAPK signaling pathway	3.03E-10	hsa05200:Pathways in cancer	6.86E-15
hsa05200:Pathways in cancer	3.78E-09	hsa04010:MAPK signaling pathway	1.20E-13
hsa04144:Endocytosis	1.05E-07	hsa04360:Axon guidance	1.97E-09
hsa04520:Adherens junction	9.67E-07	hsa04144:Endocytosis	4.43E-09
hsa04720:Long-term potentiation	6.64E-06	hsa04910:Insulin signaling pathway	5.85E-09
hsa04722:Neurotrophin signaling pathway	7.81E-06	hsa05210:Colorectal cancer	1.16E-08
hsa05211:Renal cell carcinoma	1.26E-05	hsa04722:Neurotrophin signaling pathway	2.56E-08
hsa04510:Focal adhesion	1.43E-05	hsa04810:Regulation of actin cytoskeleton	9.24E-08
hsa05220:Chronic myeloid leukemia	1.79E-05	hsa04510:Focal adhesion	1.78E-07
hsa04660:T cell receptor signaling pathway	2.29E-05	hsa05220:Chronic myeloid leukemia	4.16E-07
hsa04360:Axon guidance	2.30E-05	hsa05211:Renal cell carcinoma	5.67E-07
hsa04810:Regulation of actin cytoskeleton	3.10E-05	hsa05214:Glioma	1.11E-06
hsa04350:TGF-beta signaling pathway	5.33E-05	hsa04520:Adherens junction	3.04E-06
hsa05212:Pancreatic cancer	1.95E-04	hsa05212:Pancreatic cancer	4.32E-06
hsa05215:Prostate cancer	2.21E-04	hsa04310:Wnt signaling pathway	4.49E-06
hsa04012:ErbB signaling pathway	3.57E-04	hsa04720:Long-term potentiation	9.06E-06
hsa05218:Melanoma	4.13E-04	hsa05223:Non-small cell lung cancer	1.12E-05
hsa05210:Colorectal cancer	4.63E-04	hsa04350:TGF-beta signaling pathway	1.21E-05
hsa04310:Wnt signaling pathway	5.17E-04	hsa04930:Type II diabetes mellitus	2.20E-05
hsa05214:Glioma	0.001146	hsa05215:Prostate cancer	2.28E-05
hsa04666:Fc gamma R-mediated phagocytosis	0.001677	hsa04916:Melanogenesis	2.64E-05
hsa05414:Dilated cardiomyopathy	0.002158	hsa04660:T cell receptor signaling pathway	5.26E-05
hsa05219:Bladder cancer	0.002664	hsa04150:mTOR signaling pathway	5.53E-05
hsa04912:GnRH signaling pathway	0.002784	hsa05218:Melanoma	7.69E-05
hsa05410:Hypertrophic cardiomyopathy (HCM)	0.003003	hsa04012:ErbB signaling pathway	8.28E-05
hsa04916:Melanogenesis	0.00327	hsa05217:Basal cell carcinoma	4.94E-04
hsa05014:Amyotrophic lateral sclerosis (ALS)	0.005168	hsa05216:Thyroid cancer	6.56E-04
hsa04120:Ubiquitin mediated proteolysis	0.006743	hsa05219:Bladder cancer	0.001106
hsa04114:Oocyte meiosis	0.008262	hsa04020:Calcium signaling pathway	0.001243
hsa04330:Notch signaling pathway	0.008822	hsa04912:GnRH signaling pathway	0.001258
		hsa05213:Endometrial cancer	0.001383
		hsa04120:Ubiquitin mediated proteolysis	0.001384
		hsa04114:Oocyte meiosis	0.001741
		hsa04914:Progesterone-mediated oocyte maturation	0.001805
		hsa04270:Vascular smooth muscle contraction	0.002534
		hsa04666:Fc gamma R-mediated phagocytosis	0.002807
		hsa05414:Dilated cardiomyopathy	0.003088
		hsa05222:Small cell lung cancer	0.00502
		hsa05221:Acute myeloid leukemia	0.006889

The common pathways are underlined.

### Specific differentially expressed miRNAs

**Figure 4:**
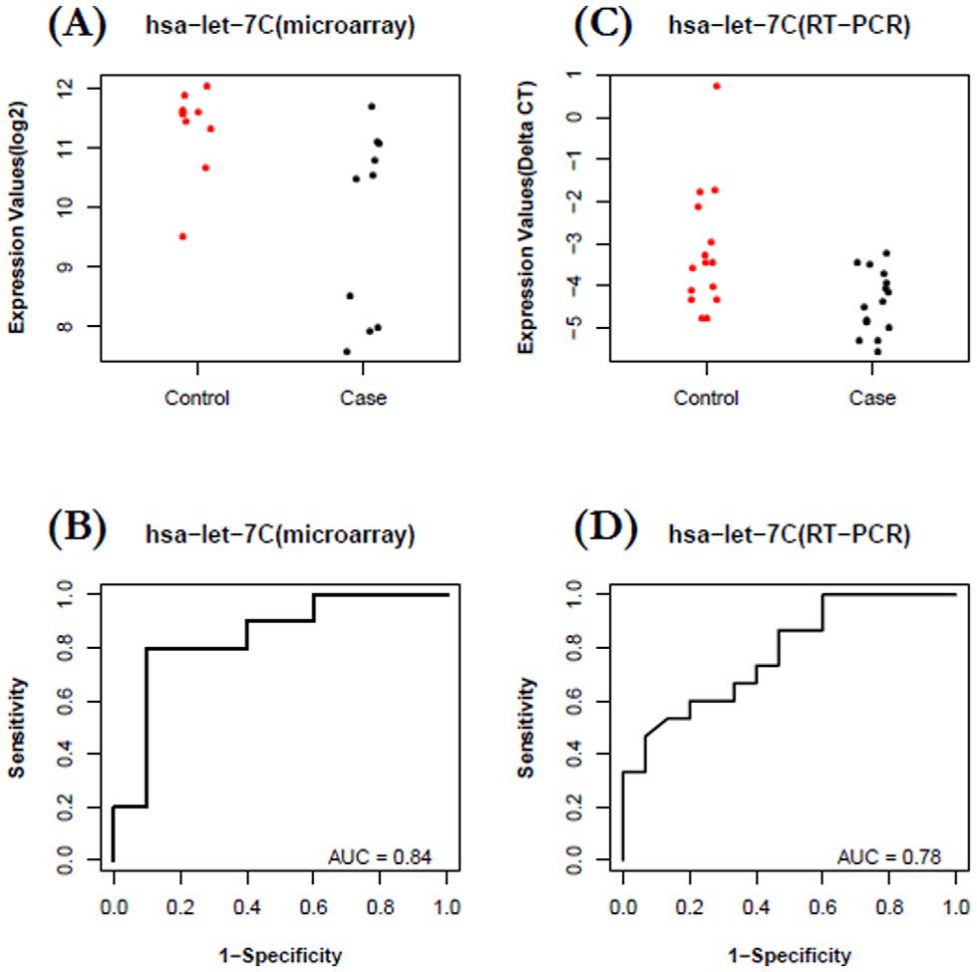
Decreased plasma levels of *let-7c* for patients with breast cancer versus healthy controls in CA group. A–B) Data from microarray profiling of 20 participants: The fold change of *let-7c* in case relative to control is −3.0 (P = 0.015, AUC  = 0.84). C–D) Data from RT-qPCR validation in an independent set of 30 participants: The fold change of *let-7c* in case relative to control is −1.9 (P = 0.01, AUC  = 0.78).

**Figure 5:**
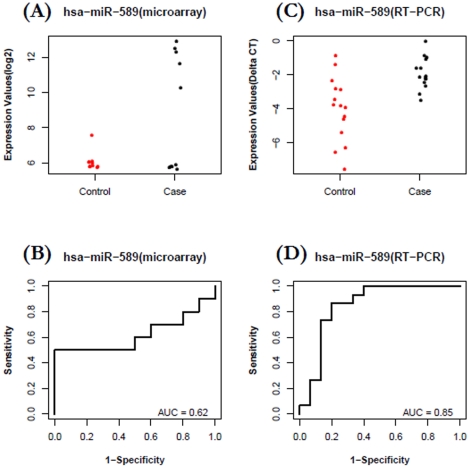
Increased plasma levels of *miR-589* for patients with breast cancer versus healthy controls in CA group. A–B) Data from microarray profiling of 20 participants: The fold change of *miR-589* in case relative to control is 6.9 (P = 0.0131, AUC  = 0.62). C–D) Data from RT-qPCR validation in an independent set of 30 participants: The fold change of *miR-589* in case relative to control is 3.3 (P = 0.0009, AUC  = 0.85).

The expression levels of miRNA were confirmed with a Taqman-based RT-qPCR. *Let-7c* and *miR-589* were selected to be validated in CA group and miR-425* and let-7d* were selected to be validated in AA group. For the ***CA group***, plasma levels of *let-7c* were, in general, lower in cases than in controls (Fig 4A) based on microarray profiling (FC = −3.0, P = 0.015). The expression data of *let-7c* in case and control groups were then used to build a ROC plot (Fig 4B), which reflects reasonable separation between the two groups (AUC = 0.84). To confirm the pattern of *let-7c* is reproducible in independent participant cohorts, we performed a validation study using 30 independent samples (15 cases vs. 15 controls). Taqman based miRNA RT-qPCR assays were used to quantify the levels of plasma miRNAs in the validation study. As shown in Figure 4C–D, a similar separation pattern was observed in this independent cohort (FC  = −1.9, P = 0.01, AUC = 0.78). *MiR-589* was identified from the microarray study with up-regulation pattern for cases over controls in CA group (Fold-Change = 6.9, p = 0.013). However, the separation is less obvious with an AUC value of 0.62 (Figure 5A–B), which ranked it as a relatively weak candidate in our microarray-based discovery phase. To further explore its biomarker potential, Taqman based miRNA RT-qPCR assays were used to quantify its expression level in the 30 independent samples (15 cases vs. 15 controls) described above. The RT-qPCR results show that this microRNA is indeed characterized by an up-regulation (FC = 3.3, P = 0.0009), and stronger separation is shown with an AUC value of 0.85 (Figure 5C–D).

**Figure 6:**
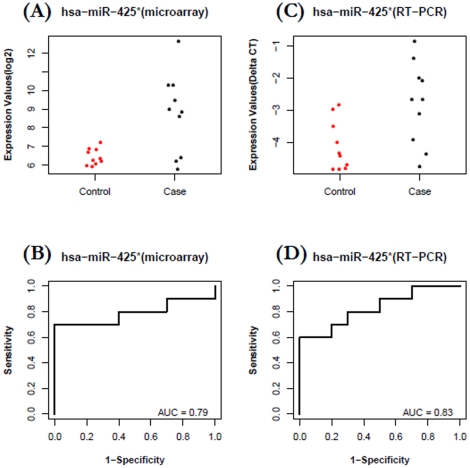
Increased plasma levels of miR-425* for patients with breast cancer versus healthy controls in AA group. A–B) Data from microarray profiling of 20 participants: The fold change of miR-425* in case relative to control is 5.0 (P = 0.00328, AUC  = 0.79). C–D) Data from RT-qPCR validation in the same 20 participants: The fold change of miR-425* in case relative to control is 3.3 (P = 0.01226, AUC  = 0.83).

**Figure 7:**
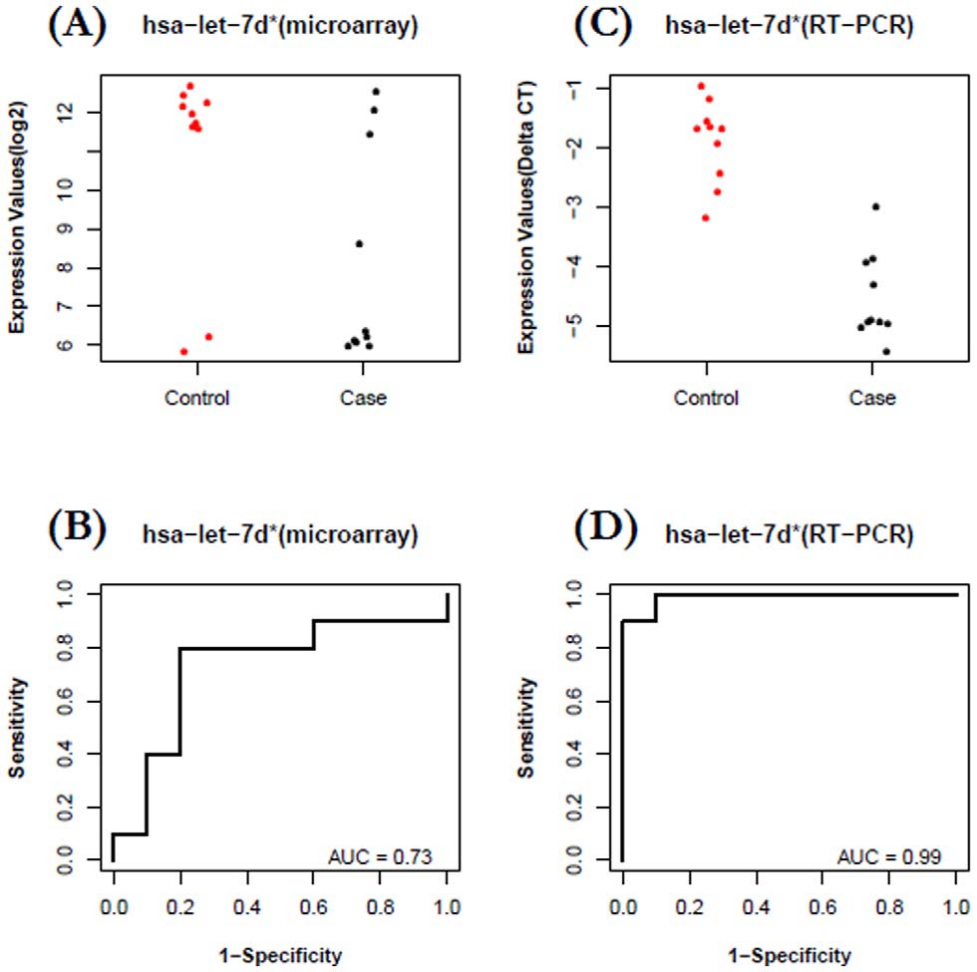
Decreased plasma levels of let-7d* for patients with breast cancer versus healthy controls in AA group. A–B) Data from microarray profiling of 20 participants: The fold change of let-7d* in case relative to control is −6.6 (p = 0.03063, AUC = 0.73). C-D) Data from RT-qPCR validation in the same 20 participants: The fold change of let-7d* in case relative to control is −9.4 (P = 1.6e-7, AUC  = 0.99).

For the ***AA group***, plasma levels of miR-425* (the minor form of *hsa-miR-425*) were, in general, higher in cases compared with controls (Fig 6A) based on microarray profiling (FC = 5.0, P = 0.00328). The expression data of miR-425* in case and control groups were used to build a ROC plot (Fig 6B), which reflects reasonable separation between the two groups (AUC = 0.79). To check the accuracy of miR-425* expression pattern obtained from microarray study, we used Taqman based miRNA RT-qPCR assays as a golden-standard platform to quantify the levels of plasma miRNAs in the same 20 samples. As shown in Figure 6C–D, a similar separation pattern was observed (FC  = 3.3, P = 0.01226, AUC  = 0.83). Let-7d* was identified from the microarray study with down-regulation pattern in cases over controls of AA group (FC  = -6.6, p = 0.03063, AUC = 0.73). Our RT-qPCR measurements in the same 20 samples confirmed the microarray results, showing that this microRNA is indeed down-regulated (FC = −9.4, P = 1.6–7), and a close-to-perfect separation is shown with an AUC of 0.99 (Figure 7). While we found that microarray and RT-qPCR are consistent in capturing the overall expression pattern of circulating miRNA (*i.e.*, up or down), the choice of different technology might result in different sensitivity and specificity (*e.g.*, AUC of 0.73 from microarray *vs.* AUC of 0.99 from RT-qPCR for let-7d*).

## Discussion

This is the first report of a comprehensive interrogation of circulating miRNAs in breast cancer patients and healthy controls. Our results demonstrate that circulating miRNAs in plasma can potentially serve as novel minimally invasive biomarkers for early detection of breast cancer. We found that 31 miRNAs were significantly differentially expressed between CA cases and CA controls and 18 miRNAs were significantly differentially expressed between AA cases and AA controls. Using selected miRNAs from those differentially expressed miRNAs, breast cancer cases and healthy controls can be discriminated with reasonable specificity and sensitivity. Intriguingly, there are only two differentially expressed miRNAs overlapping between CA and AA study subjects, suggesting potential racial differences in circulating miRNA expression. In addition, *in silico* pathway-based exploratory analysis predicted that these differentially expressed circulating miRNAs might affect critical pathways conducive to breast cancer formation and progression, a potentially important mechanism warranting further investigations.

In our study, 17 out of 31 differentially expressed miRNAs were up-regulated in CA study subjects and 9 out of 18 differentially expressed miRNAs were up-regulated in AA study subjects. Several of these up-regulated miRNAs have been reported to play an important role in carcinogenesis. For example, miR-425*, a minor form of *miR-425*, was significantly up-regulated in AA breast cancer patients compared to AA healthy controls. Although the role of miR-425* in human cancer is not clear, *miR-425* was reported to be altered in cancer cell lines and tumor tissues [36]–[37]. *miR-425* resides at 3p21, next to *miR-191* precursors embedded in the 1st intron of *DALRD3* gene. Significant abundance and co-expression of *miR-191* and *miR-425* were demonstrated in various *cancer* cell lines [36]. *miR-425* is reported to provide downregulation of ncRNA pathway via direct targeting of *DICER1* expression. *miR-302b*, which was significantly up-regulated in CA breast cancer patients compared to CA healthy controls, belongs to a panel of 12 miRNAs which are associated with inflammatory breast cancer [38]. *miR-302* family has been demonstrated to directly regulate p21. In human mammary epithelial cells, overexpression of *miR-302b* could modulate the activity of p21 and consequently alter the oncogenic phenotypes. In addition to up-regulated circulating miRNAs, we also observed a similar number of down-regulated circulating miRNAs. For example, we found the levels of circulating *let-7c* were significantly higher in healthy controls than the cases. This is consistent with the notion that *let-7* family acts as tumor suppressor genes in breast tumors. Recent studies have found that let family, especially *let-7a*, *7b* and *7c*, could inhibit cell proliferation and subsequently induce apoptosis in MCF-7 breast cancer cell lines by directly regulating ER-α [39].

The observation of decreased levels of circulating miRNAs in breast cancer patients raises an interesting question concerning the origin of circulating miRNAs and their potential functions in breast tumorigenesis. Obviously, our observation cannot be explained by the popular hypothesis that the origin of circulating miRNAs is from tumor as a result of tumor cell death and lyses. Alternatively, it is possible that both normal and tumor cells can secrete certain miRNAs. Tumor cells may secrete miRNAs that are transferable and functional in the recipient cells. However, the biological significance of such actions is still unclear. Our observations fit generally with this alternative hypothesis. However, it is clear that further functional experiment studies are needed to solve the question of the origin of circulating miRNAs.

In a previous breast cancer study, Heneghan *et al* [26] surveyed a small panel of 7 candidate miRNAs in whole blood RNAs from 148 breast cancer patients and 44 age-matched and disease free controls. They found the expression of *miR-195* was significantly higher in breast cancer patients than healthy controls (P<0.001). In addition, they found the expression of *let-7a*, which is well-regarded as a reliable endogenous control for analysis of miRNA in breast cancer, was increased over 5-fold in breast cancer patients compared to healthy controls (P<0.001). In our study, we did not observe differential expression for *miR-195* (P = 0.169) or *let-7a* (P = 0.106) between cases and controls. The discrepancy between two studies might be due to different study materials. The Heneghan study used whole blood for detection of miRNAs, while we used plasma for our analyses. Whole blood contains different types of cells, so miRNAs detected might be circulating miRNAs as well as cellular miRNAs from additional cells types. The discrepancy might also reflect the heterogeneity of breast cancer. Different molecular pathways are involved in different subtypes of breast cancer, with different molecular characterizations between luminal A, luminal B, and basal like subtypes. In the Heneghan study, 59% of breast cancers were stage I and II, 71% were invasive ductal cancer, and 82% were ER positive. Meanwhile, in our study, all patients had stage I and II invasive ductal cancer, and only 55% of patients were ER positive. These differences in clinical characteristics, together with the relatively small sample size used in this study, might contribute to the difference between their study and our study. In our ongoing validation study, we have significantly increased sample size and included different subtypes of breast cancer. Hopefully, this will give us an opportunity to validate our findings in this study as well as test the predictive value of *miR-195* and *let-7a*.

Another interesting observation from our study is the potential racial difference in terms of differentially expressed miRNAs between CA and AA study specimens. In the United States, breast cancer mortality is higher among AA women compared to women of European ancestry (EA). Breast cancer in AA women is characterized by earlier age at onset, later stage at diagnosis, higher nuclear grade, higher mitotic index and lower prevalence of estrogen receptor (ER) and/or progesterone receptor (PR) expression compared with EA women [40]. However, whether the genetic alterations leading to breast carcinogenesis are different between AA and CA breast cancer patients is not clear. More intriguingly, we found that the *in silico* predicted biological pathways were similar between CA and AAs although their miRNA expression profiles were different. This is consistent with the clinical observations that the course of breast cancer may be characterized by certain common pathways and the balance between tumor and host traits influences the pace of the common pathways. However, it should be emphasized that our observations are exploratory in nature and need to be assessed in further studies with larger sample size. More importantly, functional experiments are required to verify and establish the causal association between differentially expressed miRNAs and the predicted pathways.

One limitation of this study is the relatively small sample size which does not provide us enough power to assess relationships between levels of circulating miRNAs and clinical characteristics. To achieve an 80% power to detect a gene expression difference of 2-fold and above, we will need 18 pairs of samples, assuming a set of 1,145 human miRNA genes on the microarray, a standard deviation of 0.7, 1 expected false positive, and an adjusted *P-value* of 0.001 (http://bioinformatics.mdanderson.org/MicroarraySampleSize).

Also, as we observed in the study, there might be unknown nc-RNAs existing in the circulation, which cannot be studied using microarray analysis relying on pre-designed probes. Some of these nc-RNAs might have important predictive values and significant functional roles in breast carcinogenesis. The rapidly developing massive parallel sequencing technology is not dependent on any prior probe information, instead providing information about all known microRNAs in the sample and allowing for discovery of novel microRNAs [35]. In our data, we found that novel miRNAs constitute ∼15% of identified differentially expressed miRNAs. Nevertheless, this is the first genome-wide study to comprehensively survey the circulating miRNAs in breast cancer patients and healthy controls. The results presented here show significantly altered circulating levels of certain miRNAs in breast cancer patients compared with healthy controls. Due to the genetic and clinical heterogeneity of breast cancer, some of the separation is not perfect. Therefore, the data should be interpreted with caution. Future large studies and advanced technologies are warranted to confirm our findings and further explore the existing potential of circulating miRNAs to be utilized clinically as novel biomarkers for breast cancer.

## Supporting Information

Table S1 (0.03 MB DOC)

Table S2 (0.05 MB DOC)

Table S3 (0.04 MB DOC)

Table S4 (0.05 MB DOC)
