# Supplementary material for: A Pilot Study of Circulating miRNAs as Potential Biomarkers of Early Stage Breast Cancer
Source: PLoS One. 2010 Oct 29;5(10):e13735. doi: 10.1371/journal.pone.0013735 (PMC2966402; doi:10.1371/journal.pone.0013735)
Supplement: Table S1 — (0.03 MB DOC) [file pone.0013735.s001.doc]

**Supplementary Table 1** Summary of the number of miRNAs detected (with detection P value <0.05) in specimens from AA and CA participants, respectively.

| # of detected miRNAs  (P<0.05) | African American  10 vs. 10 | | Caucasian American  10 vs. 10 | |
| --- | --- | --- | --- | --- |
|  | Case | Control | Case | Control |
| In at least 1 patients | 878 | 865 | 873 | 886 |
| In at least 5 patients | 495 | 475 | 503 | 518 |
| In at least 10 patients | 214 | 205 | 278 | 266 |
