# Supplementary material for: A Pilot Study of Circulating miRNAs as Potential Biomarkers of Early Stage Breast Cancer
Source: PLoS One. 2010 Oct 29;5(10):e13735. doi: 10.1371/journal.pone.0013735 (PMC2966402; doi:10.1371/journal.pone.0013735)
Supplement: Table S2 — (0.05 MB DOC) [file pone.0013735.s002.doc]

**Supplementary Table 2** Expression microarray and RT-qPCR data of selected miRNA genes (miR-425* and let-7d*) in specimens from 20 AA participants (10 controls and 10 cases). The microarray data is the log2 transformed, quantile normalized expression intensity, and the RT-qPCR is the delta Ct value (miR-16 - miRNA of interest).

|  | | Microarray | | | RT-qPCR | |
| --- | --- | --- | --- | --- | --- | --- |
| Sample Status* | hsa-miR-425 | | hsa-let-7d | hsa-miR-425 | | hsa-let-7d |
| 0 | 5.95 | | 6.19 | -4.42 | | -2.44 |
| 0 | 6.83 | | 12.15 | -4 | | -1.65 |
| 0 | 6.37 | | 11.95 | -4.82 | | -1.55 |
| 0 | 6.87 | | 12.47 | -2.84 | | -0.94 |
| 0 | 6.69 | | 12.71 | -3.51 | | -1.16 |
| 0 | 7.2 | | 11.72 | -4.7 | | -1.67 |
| 0 | 6.05 | | 11.64 | -4.83 | | -1.69 |
| 0 | 5.92 | | 5.85 | -2.97 | | -1.94 |
| 0 | 6.19 | | 12.25 | -4.34 | | -2.74 |
| 0 | 6.24 | | 11.59 | -4.83 | | -3.17 |
| 1 | 8.85 | | 8.59 | -3.13 | | -4.31 |
| 1 | 9.44 | | 12.05 | -2.66 | | -3.01 |
| 1 | 9 | | 6.11 | -2.66 | | -3.93 |
| 1 | 10.26 | | 6.21 | -2 | | -4.9 |
| 1 | 12.65 | | 12.55 | -2.08 | | -4.93 |
| 1 | 10.26 | | 6.36 | -0.85 | | -3.88 |
| 1 | 8.59 | | 6.06 | -1.38 | | -5.02 |
| 1 | 5.79 | | 5.98 | -3.92 | | -4.95 |
| 1 | 6.19 | | 11.42 | -4.36 | | -4.96 |
| 1 | 6.39 | | 5.98 | -4.77 | | -5.43 |

*: 0 means control while 1 means case.
