# Supplementary material for: A Pilot Study of Circulating miRNAs as Potential Biomarkers of Early Stage Breast Cancer
Source: PLoS One. 2010 Oct 29;5(10):e13735. doi: 10.1371/journal.pone.0013735 (PMC2966402; doi:10.1371/journal.pone.0013735)
Supplement: Table S3 — (0.04 MB DOC) [file pone.0013735.s003.doc]

**Supplementary Table 3** Expression microarray data of selected miRNA genes (miR-589 and let-7c) in specimens from 20 CA participants (10 controls and 10 cases). The microarray data is the log2 transformed, quantile normalized expression intensity.

| Sample Status* | hsa-miR-589 | hsa-let-7c |
| --- | --- | --- |
| 0 | 5.79 | 10.68 |
| 0 | 5.83 | 11.62 |
| 0 | 6.06 | 11.45 |
| 0 | 6 | 11.57 |
| 0 | 5.81 | 11.63 |
| 0 | 6.02 | 12.06 |
| 0 | 5.73 | 9.52 |
| 0 | 5.78 | 11.31 |
| 0 | 7.56 | 11.9 |
| 0 | 6.1 | 11.6 |
| 1 | 5.88 | 10.8 |
| 1 | 5.81 | 10.55 |
| 1 | 10.26 | 7.9 |
| 1 | 5.75 | 7.97 |
| 1 | 12.92 | 8.51 |
| 1 | 12.49 | 10.47 |
| 1 | 12.29 | 11.09 |
| 1 | 11.64 | 11.08 |
| 1 | 5.66 | 11.72 |
| 1 | 5.79 | 7.58 |

*: 0 means control while 1 means case.
