# Supplementary material for: A Pilot Study of Circulating miRNAs as Potential Biomarkers of Early Stage Breast Cancer
Source: PLoS One. 2010 Oct 29;5(10):e13735. doi: 10.1371/journal.pone.0013735 (PMC2966402; doi:10.1371/journal.pone.0013735)
Supplement: Table S4 — (0.05 MB DOC) [file pone.0013735.s004.doc]

**Supplementary Table 4** RT-qPCR data of selected miRNA genes (miR-589 and let-7c) in specimens from 30 CA participants (15 controls and 15 cases). The RT-qPCR is the delta Ct value (miR-16 - miRNA of interest).

| Sample Status* | hsa-miR-589 | hsa-let-7C |
| --- | --- | --- |
| 0 | -1.41 | -4.04 |
| 0 | -2.8 | -4.13 |
| 0 | -3.84 | -3.46 |
| 0 | -6.29 | -2.95 |
| 0 | -7.53 | -1.8 |
| 0 | -2.33 | -4.79 |
| 0 | -3.78 | -3.59 |
| 0 | -4.59 | -3.44 |
| 0 | -3.47 | 0.75 |
| 0 | -5.39 | -4.34 |
| 0 | -6.55 | -3.28 |
| 0 | -0.87 | -2.12 |
| 0 | -2.88 | -1.72 |
| 0 | -3.91 | -4.32 |
| 0 | -4.43 | -4.79 |
| 1 | -2.24 | -4.5 |
| 1 | -3.15 | -5.55 |
| 1 | -3.51 | -3.48 |
| 1 | -1.09 | -4.87 |
| 1 | -0.01 | -4.16 |
| 1 | -2.23 | -5.3 |
| 1 | -1.63 | -4.36 |
| 1 | -2.43 | -4.99 |
| 1 | -0.96 | -3.25 |
| 1 | -0.9 | -3.92 |
| 1 | -2.15 | -3.72 |
| 1 | -2.13 | -4.84 |
| 1 | -1.6 | -3.46 |
| 1 | -2.09 | -5.29 |
| 1 | -2.67 | -4.07 |

*: 0 means control while 1 means case.
